# RNA-Seq Meta-analysis identifies genes in skeletal muscle associated with gain and intake across a multi-season study of crossbred beef steers

**DOI:** 10.1186/s12864-018-4769-8

**Published:** 2018-06-04

**Authors:** Brittney N. Keel, Christina M. Zarek, John W. Keele, Larry A. Kuehn, Warren M. Snelling, William T. Oliver, Harvey C. Freetly, Amanda K. Lindholm-Perry

**Affiliations:** 1 USDA, ARS, U.S. Meat Animal Research Center, Clay Center, NE 68933 USA; 2 Current Affiliation: UT Southwestern Medical Center, Dallas, TX 75390 USA

**Keywords:** Beef cattle, Differential expression, Feed efficiency, RNA-Seq, Meta-analysis, Transcriptome

## Abstract

**Background:**

Feed intake and body weight gain are economically important inputs and outputs of beef production systems. The purpose of this study was to discover differentially expressed genes that will be robust for feed intake and gain across a large segment of the cattle industry. Transcriptomic studies often suffer from issues with reproducibility and cross-validation. One way to improve reproducibility is by integrating multiple datasets via meta-analysis. RNA sequencing (RNA-Seq) was performed on longissimus dorsi muscle from 80 steers (5 cohorts, each with 16 animals) selected from the outside fringe of a bivariate gain and feed intake distribution to understand the genes and pathways involved in feed efficiency. In each cohort, 16 steers were selected from one of four gain and feed intake phenotypes (*n* = 4 per phenotype) in a 2 × 2 factorial arrangement with gain and feed intake as main effect variables. Each cohort was analyzed as a single experiment using a generalized linear model and results from the 5 cohort analyses were combined in a meta-analysis to identify differentially expressed genes (DEG) across the cohorts.

**Results:**

A total of 51 genes were differentially expressed for the main effect of gain, 109 genes for the intake main effect, and 11 genes for the gain x intake interaction (P_corrected_ < 0.05). A jackknife sensitivity analysis showed that, in general, the meta-analysis produced robust DEGs for the two main effects and their interaction. Pathways identified from over-represented genes included mitochondrial energy production and oxidative stress pathways for the main effect of gain due to DEG including *GPD1*, *NDUFA6*, *UQCRQ*, *ACTC1*, and *MGST3*. For intake, metabolic pathways including amino acid biosynthesis and degradation were identified, and for the interaction analysis the pathways identified included GADD45, pyridoxal 5’phosphate salvage, and caveolar mediated endocytosis signaling.

**Conclusions:**

Variation among DEG identified by cohort suggests that environment and breed may play large roles in the expression of genes associated with feed efficiency in the muscle of beef cattle. Meta-analyses of transcriptome data from groups of animals over multiple cohorts may be necessary to elucidate the genetics contributing these types of biological phenotypes.

**Electronic supplementary material:**

The online version of this article (10.1186/s12864-018-4769-8) contains supplementary material, which is available to authorized users.

## Background

Feed costs are the major component of production costs in the beef cattle industry, accounting for 55–75% of total production costs [1–3]. One way to potentially reduce these costs is to improve efficiency of beef cattle. Feed intake and weight gain are two measureable component phenotypes that are often used to characterize the feed efficiency of an animal. Dry matter intake (DMI), residual feed intake (RFI), average daily gain (ADG), and feed conversion ratio (FCR) have been shown to be under genetic control, with heritabilities estimated between 0.2 and 0.5 [3, 4], indicating that these traits could be improved through selection.

National cattle evaluations routinely include information from gain to predict genetic merit for growth. However, individual feed intake measurements are difficult and expensive to obtain. Hence, information is lacking for generation of predictions of total genetic merit for feed intake and efficiency in major cattle breeds. An improved understanding of the regulation of genes underlying efficiency could improve the effectiveness of selection for efficiency as well as significantly reduce the cost of doing so.

Skeletal muscle is responsible for approximately 25% of an animal’s requirements for maintenance energy due to its size and involvement with energy production [5, 6]. Bovine skeletal muscle has been the subject of several targeted gene expression studies, attributable to its link with feed efficiency via roles in mitochondrial energy production [5–8]. To date, a small number of studies have examined the bovine skeletal muscle transcriptome and its role in feed efficiency [9, 10]; however, none of these encompass more than one contemporary group (or cohort) of cattle. Weber et al. [10] conducted a study comparing differential gene expression of low and high RFI animals using RNA sequencing (RNA-Seq) from skeletal muscle of 16 Angus steers sired by one high RFI bull and one low RFI bull. Genes involved in fat deposition, immune/inflammatory function, and cell damage were identified as differentially expressed among these animals. Another skeletal muscle transcriptome study was performed by Guo et al. [11] on 48 Brahman steers that identified cell cycle, extracellular matrix and fat deposition genes involved in gain.

Non-reproducibility of results is a major problem in high-dimensional experiments such as gene expression analysis, where thousands of hypotheses are being tested simultaneously [12]. Due to the cost of sequencing, RNA-Seq experiments are typically performed on a small number of biological replicates, which limits their power for detecting differentially expressed genes (DEG). Additionally, variability between studies due to technical differences (e.g., sample preparation, library protocols, batch effects) as well as biological differences (e.g. environmental and genetic effects) also contributes to reproducibility issues. One way to improve reproducibility is by integrating multiple datasets via meta-analysis. Meta-analysis procedures have been previously shown to produce results that are more likely to be valid in independent datasets [13–15].

A major aim of this study was to discover differentially expressed genes that will be robust across a large segment of the cattle industry. As such, crossbred steers from a population representing 19 *Bos taurus* and *Bos indicus* breeds were used in this study. Many of the previous studies in cattle have used RFI as the phenotype, which is the difference between actual and expected feed intake. The calculation of RFI is based on several factors including, ADG, average daily feed intake (ADFI), growth rate, weight, and efficiency of growth [16]. In order to gain a more detailed understanding of the role of DEGs in feed efficiency we incorporated two components of RFI: ADG and ADFI. Samples were collected from animals in five separate feeding trials, and a meta-analysis procedure was implemented to identify DEGs across the cohorts that could be attributed to gain and feed intake, as well as the gain by intake interaction.

## Methods

### Animal care and use

The U.S. Meat Animal Research Center (USMARC) Animal Care and Use Committee reviewed and approved all animal procedures. The procedures for handling cattle complied with the Guide for the Care and Use of Agricultural Animals in Agricultural Research and Teaching [17].

### Population

A total of 80 steers, originating from the continuous phase of the USMARC Germplasm Evaluation project [18], were used in this study. The Germplasm Evaluation project is a breeding program intended to develop several populations of cattle with a high percentage of top U.S. beef breeds, including Angus, Beefmaster, Brahman, Brangus, Brown Swiss, Charolais, Chiangus, Gelbvieh, Hereford, Limousin, Maine Anjou, Red Angus, Romosinuano, Bonsmara, South Devon, Salers, Santa Gertrudis, Shorthorn, and Simmental.

### Gain and feed intake

Crossbred steers were collected as groups of 16 animals from 5 cohorts. Feed intake and body weight gain were measured on steers from 2012 to 2014 (Additional file 1(A)). Steers were 350 ± 54 days of age at the beginning of the feeding trial and trials lasted 64–92 days, during which they were fed a corn-based finishing diet (Tables 1 and 2). Steers were housed in pens (15.2 × 45.7 m) in a facility that was equipped with an Insentec Roughage Intake Control Feeding System (Insentec B.V., Marknesse, The Netherlands). Each pen housed approximately 50 steers and had 8 feed bunks. Steers had access to all of the feed bunks in the pen and the bunks measured individual feed intake. Feeders and waterers were located inside an open-sided barn. The barn was 4.8 m high at the front and covered 162 m^2^ of the pen.

**Table 1:** Length of time and diets used for feed trials

Year Born	Time of Slaughter	Start Date	End Date	Days on Study	Diet^a^
2011	Spring 2012	04/11/12	06/14/12	64	ME02
2011	Fall 2012	07/10/12	10/10/12	92	ME01
2012	Spring 2013	04/16/13	06/19/13	64	ME01
2012	Fall 2013	07/24/13	10/16/13	84	TM01
2013	Fall 2014	07/29/14	10/15/14	78	TM01

^a^Diet composition provided in Table 2

**Table 2:** Composition of diets used in feed trials

	ME01	ME02	TM01
Dry rolled corn	57.35%	0%	57.35%
Ground alfalfa hay	8%	8%	8%
High moisture corn	0%	57.75%	0%
Steakmaker®^a^	4.25%	4.25%	0%
Steakmaker with Tylan^b^	0%	0%	4.25%
Urea	0.4%	0%	0.4%
Wet distiller’s grains with solubles	30%	30%	30%

^a^Manufactured by Land O’Lakes (Arden Hills, MN)

^b^Tylan manufactured by Elanco Animal Health (Greenfield, IN)

**Fig. 1:**
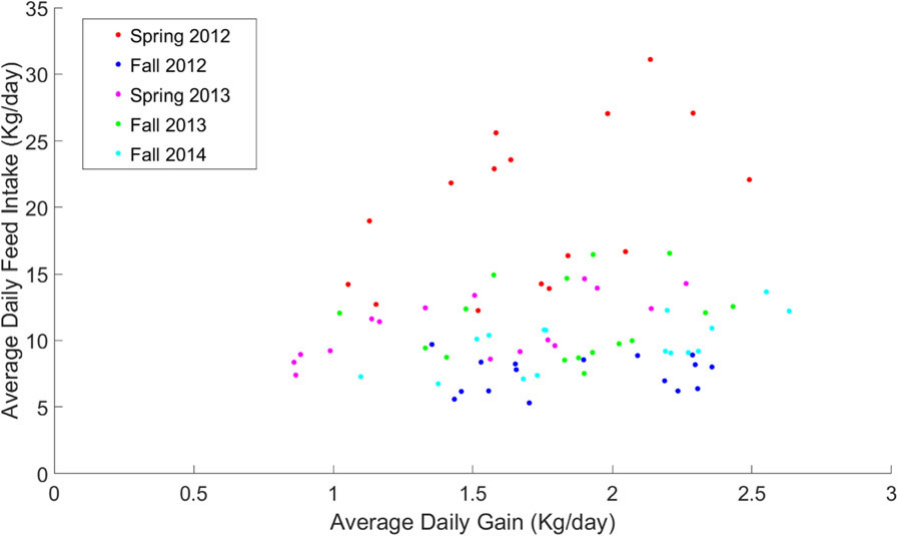
Total gain versus total dry matter intake over the trial period was plotted for all animals (*n* = 80) used in this study. Cohorts are represented by the color of the dots

Steers were weighed on the first two and last two days and every three weeks during the study. Total gain was calculated by regressing body weight on days on study using a quadratic polynomial. Average daily gain was calculated as total body weight gained divided by days on study. Sixteen steers were selected from each cohort by ranking them based on their standardized distance from the bivariate mean (of ADG and ADFI) assuming a bivariate normal distribution with calculated correlation between ADG and ADFI. The four steers with the greatest deviation from the bivariate mean in each Cartesian quadrant were sampled. This resulted in the selection of 16 steers, 4 steers from each of 4 quadrants: the high gain-high intake quadrant, the high gain-low intake quadrant, the low gain-high intake quadrant, and the low gain-low intake quadrant (Fig. 1). To ensure that breed composition was not confounded with quadrant within cohort, steers were selected to ensure that each quadrant had multiple breeds represented. In the event a sire breed was over represented within a quadrant, a steer with the next highest ranking of a different breed was selected. Breed percentages for the selected animals in each quadrant in each cohort are shown in Additional file 1(B). Animals with medical or health issues that might affect gain or intake were also excluded from selection (Additional file 1(C)). A summary of the gain and feed intake means, minimums, and maximums for each quadrant in each cohort is provided in Table 3.

**Table 3:** Summary statistics for ADG and ADFI (Kg/day) in the animals selected from each of the cohorts

	Mean ADG	Min. ADG	Max. ADG	Mean ADFI	Min. ADFI	Max. ADFI
Spring 2012						
High Gain-High Intake	2.22	1.98	2.49	26.83	22.08	31.10
Low Gain-High Intake	1.55	1.42	1.64	23.48	21.83	25.61
Low Gain-Low Intake	1.21	1.05	1.52	14.54	12.25	18.97
High Gain-Low Intake	1.85	1.75	2.05	15.29	13.91	16.65
Fall 2012						
High Gain-High Intake	2.26	2.09	2.36	8.49	8.00	8.91
Low Gain-High Intake	1.68	1.53	1.90	8.24	7.8	8.55
Low Gain-Low Intake	1.54	1.43	1.70	5.81	5.30	6.20
High Gain-Low Intake	2.02	1.35	2.31	7.31	6.19	9.70
Spring 2013						
High Gain-High Intake	2.06	1.90	2.26	13.82	12.40	14.65
Low Gain-High Intake	1.28	1.14	1.51	12.22	11.41	13.40
Low Gain-Low Intake	0.90	0.86	0.99	8.48	7.39	9.22
High Gain-Low Intake	1.70	1.56	1.79	9.35	8.60	10.04
Fall 2013						
High Gain-High Intake	2.23	1.93	2.43	14.40	12.09	16.53
Low Gain-High Intake	1.48	1.02	1.84	13.51	12.06	14.93
Low Gain-Low Intake	1.61	1.33	1.88	8.84	8.52	9.43
High Gain-Low Intake	1.98	1.90	2.07	9.08	7.51	9.98
Fall 2014						
High Gain-High Intake	2.43	2.20	2.63	12.27	10.92	13.67
Low Gain-High Intake	1.65	1.51	1.76	10.52	10.10	10.79
Low Gain-Low Intake	1.47	1.10	1.73	7.12	6.74	7.37
High Gain-Low Intake	2.24	2.19	2.31	9.13	9.06	9.19

After the feed trial ended, selected animals were comingled in a pen with ad libitum access to the same diet. Length of time between the feed trial and slaughter varied slightly by cohort: cohort 1 was 12–18 days, cohort 2 was 19–22 days, cohort 3 was 5–8 days, cohort 4 was 20–28 days, and cohort 5 was 11–14 days. Animals were allowed to consume feed and water until they were weighed on the day of slaughter and transported to the US Meat Animal Center abattoir (under 6.4 km). Cohort 5 was transported to a small commercial abattoir and processing plant approximately 20 miles away. Aside from transport location and distance, all other parameters remained the same. On each day of slaughter, four of the animals selected (one from each phenotypic group) were stunned with captive bolt, exsanguinated, and processed serially within a three-hour time frame.

### Tissue collection and RNA isolation

Tissue collection and RNA extraction were performed using the same procedures in each cohort. A longissimus dorsi sample between the sixth and seventh rib from the right side of the carcass was taken 25–30 min post exsanguination. Sample collection time frame was consistent across cohorts. The sample was diced into approximately one cubic centimeter pieces, flash frozen in liquid nitrogen, and stored at − 80 °C until RNA isolation. Muscle samples (50 to 100 mg) from the 80 animals were homogenized with a six station Omni Prep homogenizer (Omni International, Kennesaw, GA, USA) in one milliliter of Trizol. Total RNA was isolated according to the manufacturer’s protocol and was resuspended in 50–100 μL RNase-free water.

Genomic DNA was removed from the total RNA with the Qiagen RNeasy mini-kit (Valenci, CA, USA), according to the manufacturer’s protocol. The concentration of the RNA was determined with a Nanodrop 8000 spectrophotometer (Thermo Scientific, Wilmington, DE, USA). The average 260/280 ratio was 2.04, with a range of 1.95–2.13. An Agilent Bioanalyzer RNA 6000 nano kit (Santa Clara, CA, USA) was used to determine the RNA integrity number (RIN). Only samples with a RIN of 7.0 and higher were used for the RNA sequencing. The average RIN was 7.8, with a range of 7.0–8.5.

### RNA sequencing

Samples were prepared for RNA sequencing with the Illumina TruSeq Stranded mRNA High Throughput Sample kit and protocol (Illumina Inc., San Diego, CA, USA). The libraries were quantified with qRT-PCR using the NEBNext Library Quant Kit (New England Biolabs, Inc., Beverly, MA, USA) on a CFX384 thermal cycler (Bio-Rad, Hercules, CA, USA), and the quality of the library was determined with an Agilent Bioanalyzer DNA kit (Santa Clara, CA, USA). The libraries were diluted with Tris-HCL 10 mM, pH 8.5 with 0.1% Tween 20 to 10 nM samples (Teknova, Hollister, CA. USA). The libraries were pooled into eight pools of 12 libraries in each according to Illumina’s dual-index protocol (samples were submitted for sequencing with 16 libraries from another RNA-Seq study). All 80 samples were paired-end sequenced with 150 cycle high output sequencing kits for the Illumina NextSeq instrument.

### Processing RNA-Seq data

Read alignment of the RNA-Seq data was carried out as follows. First, quality of the raw paired-end sequence reads in individual fastq files was assessed using FastQC (Version 0.11.5; www.bioinformatics.babraham.ac.uk/projects/fastqc), and reads were trimmed to remove adapter sequences and low-quality bases using the Trimmomatic software (Version 0.35) [19]. The remaining reads were mapped to the UMD 3.1 genome assembly using Tophat2 (Version 2.1.1) [20], and the NCBI annotation for UMD 3.1 was used to guide the alignment. We used the HTSeq package [21] to estimate the count of uniquely mapped reads for each of the 28,451 annotated genes in the NCBI UMD 3.1 gene transfer format (GTF) file. Genes with low read counts, < 15 reads in at least 16 samples, were removed resulting in a set of 13,511 genes to be used in our downstream analysis.

### Meta-analysis of differential gene expression

Recently, generalized linear models (GLMs) have been utilized for analyzing differential gene expression in RNA-Seq experimental designs involving multiple explanatory factors [22–24]. In such a GLM, analysis of deviance (ANODEV) is used to identify DEGs associated with individual factors and their interactions. We used the following GLM with a negative binomial link function, which simultaneously considers two explanatory variables, gain (H vs. L) and intake (H vs. L), to analyze differential expression:

1 $$ Y=\kern0.5em Gain\kern0.5em +\kern0.5em Intake\kern0.5em +\kern0.5em Gain\times \kern0.5em Intake $$

We used the R package DESeq2 [23] to perform our differential expression analysis. DESeq2 uses a negative binomial distribution to model gene read counts and shrinkage estimators to estimate the per-gene negative binomial dispersion parameters.

The function nbinomLRT, which performs a likelihood-ratio test between a full and a reduced negative binomial GLM, was used to test three separate null hypotheses. Null hypothesis 1 tested whether each gene was significantly affected by gain, null hypothesis 2 tested whether each gene was significantly affected by intake, and null hypothesis 3 tested whether each gene was significantly affected by the interaction between gain and intake.

In the meta-analysis, each cohort was analyzed separately using the GLM in Eq. (1). The raw *P*-values for each gene from each of the five analyses were combined using Fisher’s method [25], which combines P-values from each experiment into one test statistic defined as

2 $$ X=\kern0.5em -2\sum \limits_{s=1}^s\ln \left({p}_{gs}\right), $$

where *p*_*gs*_ denotes the raw *P*-value obtained from gene *g* in experiment *s* and *S* is the number of experiments being combined. Under the null hypothesis, the test statistic *X* follows a χ^2^ distribution with 2*S* degrees of freedom. This test provides a meta *P*-value, and classical procedures for multiple testing correction can be applied to obtain *P*-values adjusted to control the false discovery rate. The Benjamini-Hochberg method [26] was used to correct for multiple testing. Genes with adjusted meta *P*-value ≤0.05 were considered statistically significant. DEGs associated with a main effect and also the interaction term were excluded from the main effect list of DEGs since this indicates that the main effect is dependent on the interaction term.

### Jackknife reproducibility analysis

Robustness of the results was evaluated using a jackknife sensitivity analysis; i.e. the meta-analysis procedure was repeated multiple times, each time with removal of a single cohort from the baseline group of cohorts [27].

### Functional analysis of DEGs

Functions of DEGs were determined using the PANTHER classification system (Version 11.0) [28]. Enrichment analysis of gene function was performed using PANTHER’s implementation of the binomial test of overrepresentation. Significance of gene ontology (GO) terms was assessed using the default Ensembl *Bos taurus* GO annotation as background for the enrichment analysis. Data from PANTHER was considered statistically significant at Bonferroni corrected *P* ≤ 0.05.

### QIAGEN ingenuity® pathway analysis

Ingenuity® Pathway Analysis (IPA®, QIAGEN Redwood City, CA; https://www.qiagenbioinformatics.com/products/ingenuity-pathway-analysis/) was used to deduce direct and indirect molecular relationships among differentially expressed genes for the gain main effect, intake main effect, and gain x intake interaction. Each of the data sets was imported with a Flexible Format using Gene symbol as the identifier. A core analysis was performed on genes in each set, where a *P*-value for each network is calculated according to the fit of the users set of significant genes and the size of the network. *P*-values were considered statistically significant at Benjamini-Hochberg adjusted *P* ≤ 0.05.

## Results

### Sequencing throughput, read mapping, and read counts

RNA-Seq libraries from the longissimus dorsi muscle tissue of 80 steers with divergent components of feed efficiency were sequenced. We generated over 6 billion 75-bp paired-end reads using an Illumina NextSeq instrument. The range of raw sequence reads per sample was 23.4 million to 194.7 million, with an average of 79.9 million reads per sample (Additional file 1(D)). Adapter sequences and low quality bases were trimmed with the Trimmomatic software, which resulted in approximately a 0.02% reduction in the number of reads across the 80 samples. The resulting high quality reads were mapped to the *Bos taurus* UMD 3.1 genome assembly with an average 93.4% overall mapping rate. Computing read counts for each gene and filtering out genes with low read counts resulted in a set of 13,511 genes to be used for downstream analysis.

### Meta-analysis of DEGs associated with gain and feed intake across cohorts

Selection of individuals with extreme gain and feed intake phenotypes to be used in this study was done within cohort. Figure 1 shows ADG versus ADFI over the feeding period for all animals (*n* = 80) used in this study. This plot clearly illustrates that there is a segregation of phenotypes across cohorts.

Since our goal was to identify DEGs that could explain the overall variation in gain and feed intake across all cohorts, we performed a meta-analysis of differential expression across the 5 cohorts. In this procedure differential gene expression analysis was performed independently for each cohort using the GLM shown in Eq. (1). There was variation among the cohorts in the number of genes identified as differentially expressed with *P* ≤ 0.05 after FDR correction (data not shown). For the gain main effect, there were 4, 0, 14, 10, and 0 DEGs in Cohort 1, 2, 3, 4, and 5, respectively. For the intake main effect, 0, 0, 14, 10, and 0 genes were identified as differentially expressed. The analysis of the gain by intake interaction produced 0, 0, 14, 10, and 0 DEGs.

Raw *P*-values for each gene from each individual cohort analysis were then combined using Fisher’s method. After multiple testing correction, we identified 51 significant genes for the gain main effect, 109 genes that were significant for the intake main effect, and 11 significant genes for the gain x intake interaction (Additional files 2, 3 and 4). Significant genes were inspected for consistency, defined as having the same log-fold change direction across all 5 cohorts. We found that only 4 significant genes (*LOC515676, UQCRQ, NPR3, C5H12orf5*) were consistent for the gain main effect, while 8 DEGs (*IQANK1, LOC101904159, LOC101904117, CD163, MCHR1, MFSD4, OAT, TNNI1*) were consistent for the intake main effect. No DEGs were consistent for the gain x intake interaction.

### Jackknife analysis

Robustness of the results were assessed using a jackknife sensitivity analysis, where for each term in the model five separate meta-analyses were performed each omitting a single cohort. The results are shown in Additional files 5, 6 and 7. For the gain main effect, the jackknife analyses produced similar numbers of DEGs to that of the original meta-analysis (51 DEGs): 69, 25, 17, 44, and 51 DEGs for the jackknife analysis that removed Cohort 1, 2, 3, 4, and 5, respectively (Jacknife *P* < 0.05 in Additional file 5). The number of DEGs identified in the jackknife analyses for the intake main effect varied more than for gain, with 219, 60, 56, 59, and 38 DEGs for the jackknife analysis that removed Cohort 1, 2, 3, 4, and 5, respectively (Jacknife *P* < 0.05 in Additional file 6). The jackknife analyses for the interaction effect identified 21, 16, 2, 3, and 6 DEGs for the jackknife analysis that removed Cohort 1, 2, 3, 4, and 5, respectively (Jacknife *P* < 0.05 in Additional file 7), which was highly similar to the number of DEGs identified in the full meta-analysis (11 DEGs).

For the gain main effect, there were no DEGs that were robust enough to pass all five jackknife analyses. Eleven DEGs failed only one jackknife analysis, while 19, 18, 3, and 0 DEGs failed to pass 2, 3, 4, and 5 jackknife analyses, respectively. For the intake main effect, there were 2 DEGs, *ZNF775* and *CST6,* that passed all five jackknife analyses, and there were 2 DEGs, *TMEM120A* and *LOC508916*, that failed all five jackknife analyses. Thirty DEGs failed only one jackknife analysis, while 32, 32, and 11 DEGs failed to pass 2, 3, and 4 jackknife analyses, respectively. For the interaction effect, there were no DEGs that passed all five tests and none that failed all five, while 3, 6, 1, and 1 DEGs failed to pass 1, 2, 3, and 4 tests, respectively.

### Function of DEGs

PANTHER gene ontology analysis of the DEGs indicated that genes that were significant for the gain main effect were involved in catalytic activity (47.4%), binding (31.6%), structural molecule activity (15.8%), and antioxidant activity (5.3%). No GO terms were significantly over- or under-represented in this gene set.

Similar to the gain main effect genes, genes that were significant for the intake main effect were involved in binding (38.8%), catalytic activity (36.7%), transporter activity (10.2%), receptor activity (8.2%), structural molecule activity (4.1%), and signal transducer activity (2%). Again, no GO terms were significantly over- or under-represented in this set.

Genes that were significant for the gain x intake interaction were involved in catalytic activity (50%), binding (16.7%), transporter activity (16.7%), and structural molecule activity (16.7%). Enrichment analysis of GO terms did not identify any over- or under-represented GO terms in these genes.

### Ingenuity pathway analysis (IPA)

Ingenuity® Pathway Analysis was performed in order to characterize the functional consequences of gene expression differences for the gain main effect, intake main effect, and gain x intake interaction. IPA identified thirteen significant canonical pathways for the DEGs associated with the gain main effect (Table 4). The top five canonical pathways included mitochondrial dysfunction represented by the genes *BCL2, NDUFA6, UQCRQ (P < 0.01),* glycerol-3-phosphate shuttle due to *GPD1* (*P* < 0.01), glycerol degradation I from gene *GPD1* (*P* = 0.0101), death receptor signaling from DEGs *BCL2, ACTC1* (*P* = 0.0107), and VEGF signaling due to *ACTC1, BCL2* (*P* = 0.013). Molecular and cellular functions related to these genes included amino acid metabolism, molecular transport, small molecule biochemistry, cellular growth and proliferation, and cell death and survival.

**Table 4:** Significant pathways for DEGs associated with the gain main effect identified using IPA

Pathway	*P*-value^a^	DEGs in Pathway
Mitochondrial Dysfunction	0.00296	*BCL2, NDUFA6, UQCRQ*
Glycerol-3-phosphate shuttle	0.00672	*GPD1*
Glycerol degradation I	0.0101	*GPD1*
Death receptor signaling	0.0107	*ACTC1, BCL2*
VEGF signaling	0.013	*ACTC1, BCL2*
Oxidative phosphorylation	0.0145	*NDUFA6, UQCRQ*
Phosphatidylethanolamine biosynthesis II	0.0151	*ETNK2*
Pancreatic adenocarcinoma signaling	0.0169	*BCL2, RALGDS*
Gai signaling	0.0174	*NPR3, RALGDS*
nNOS signaling in skeletal muscle cells	0.025	*SNTA1*
Glutathione redox reactions I	0.0397	*MGST3*
NRF2-mediated oxidative stress response	0.0418	*ACTC1, MGST3*
ILK signaling	0.043	*ACTC1, LIMS2*

^a^Corrected for multiple testing using Benjamini Hochberg method

For the intake main effect, IPA identified twelve significant pathways (Table 5), including 4-hydroxyproline degradation I due to *HOGA1* (*P* < 0.01), methyglyoxal degradation I because of *HAGHL* (*P* = 0.012), D-glucuronate degradation I due to *DCXR* (*P* = 0.0120), glycerol-3-phosphate shuttle because of *GPD1* (*P* = 0.016), and arginine degradation (arginase pathway) due to *OAT* (*P* = 0.016). Molecular and cellular functions related to these genes included cellular movement, cell cycle, cellular development, cellular growth and proliferation, and cell death and survival.

**Table 5:** Significant pathways for DEGs associated with the intake main effect identified using IPA

Pathway	*P*-value^a^	DEGs in Pathway
4-hydroxyproline degradation I	0.00803	*HOGA1*
Methylglyoxal degradation I	0.012	*HAGHL*
D-glucuronate degradation I	0.012	*DCXR*
Glycerol-3-phosphate shuttle	0.016	*GPD1*
Arginine degradation I (arginase pathway)	0.016	*OAT*
Arginine biosynthesis IV	0.0239	*OAT*
Proline biosynthesis II (from arginine)	0.0239	*OAT*
Arginine degradation VI (arginase 2 pathway)	0.0239	*OAT*
Glycerol degradation I	0.0239	*GPD1*
CNTF signaling	0.0267	*CNTFR, HRAS*
Citrulline biosynthesis	0.0318	*OAT*
PPARa/RXRa activation	0.0362	*CHD5, GPD1, HRAS*

^a^Corrected for multiple testing using Benjamini Hochberg method

Lastly, for the interaction effect there were four significant pathways (Table 6), GADD45 signaling due to *GADD45B* (*P* < 0.01), pyridoxal 5′-phosphate salvage pathway due to *PDXK* (*P* < 0.001), caveolar-mediated endocytosis signaling due to *ALB* (*P* = 0.0327), and ATM signaling due to *GADD45B* (*P* = 0.0368). Molecular and cellular functions related to these genes included cellular movement, amino acid metabolism, antigen presentation, carbohydrate metabolism, and cell death and survival.

**Table 6:** Significant pathways for DEGs associated with the gain by intake interaction effect identified using IPA

Pathway	*P*-value^a^	DEGs in Pathway
GADD45 signaling	0.00886	*GADD45B*
Pyridoxal 5′-phosphate salvage pathway	0.03	*PDXK*
Caveolar-mediated endocytosis signaling	0.0327	*ALB*
ATM signaling	0.0368	*GADD45B*

^a^Corrected for multiple testing using Benjamini Hochberg method

## Discussion

To date, there have been very few transcriptome meta-analyses for livestock species reported in the literature. RNA-Seq experiments, especially those performed in livestock, are routinely performed on a small number of biological replicates due to the cost of sequencing. The limited power in these studies coupled with both technical and biological variability between studies can lead to issues with reproducibility and cross-validation. Meta-analysis can help improve research findings in these types of studies by eliminating false-positive findings pertaining to experimental and design conditions. Moreover, integrating data across multiple experiments may enable extraction of deeper biological insights compared to that achieved through single-study analysis. To our knowledge, this is the first RNA-Seq study in livestock where data was collected over multiple cohorts, each cohort serving as a separate experiment, and analyzed using a meta-analysis procedure.

The purpose of this study was to identify genes differentially expressed in the muscle of beef cattle associated with gain and feed intake that will be robust across the cattle industry for selection of more feed efficient animals. There were a total of 19 breeds (plus MARC II and MARC III composites) represented in the study with all but three of them, Bonsmara, Romosinuano, and South Devon, represented in multiple cohorts and in more than one phenotypic class (for example, animals with Chiangus as a portion of their breed-of-origin are represented in the low gain-high intake class in Fall 2012 and the low gain- high intake and low gain-low intake classes in Fall 2013). Moreover, both fall and spring seasons over 3 years are represented among the five cohorts. The rationale for this design was to generate data that would include the most robust drivers of gene expression affecting feed intake and gain.

The variation among the lists of genes identified as differentially expressed by cohort in this study underscores the importance of including animals from more than one cohort of livestock to obtain biologically relevant data for complex traits. Validation of transcriptomic or proteomic data is likely to produce poor reproducibility from study to study due to the large amount of biological variation from sources that include breed and environmental factors. For this reason, we chose to measure reproducibility by replication validity rather than in independent data, such as cross-validation.

Genes passing all five jackknife analyses can be considered highly robust, as they are not dependent on any one cohort. Genes that fail multiple jackknife tests can also be interpreted as robust, where the higher number of failed tests indicates greater robustness. This interpretation can be derived as follows. If a gene fails only one jackknife test, this indicates that the meta *P*-value is being driven by the *P*-value arising from this single cohort. Hence, there may be some cohort bias for that gene. On the other hand, if a gene fails multiple jackknife tests, then the meta *P*-value is being driven by the *P*-values of multiple cohorts, i.e. there is a reduced level of cohort bias.

We saw that only two genes passed all five jackknife tests and two genes failed all five jackknife tests for the intake main effect, and none passed or failed all five tests for gain and interaction. That is, there were more highly robust genes observed for the intake main effect than the gain main effect. It has been shown that DMI is a moderately repeatable trait, while ADG exhibits low repeatability [29, 30]. In general, DEGs associated with both main effects tended to be moderately robust, with 41.1 and 41.3% of DEGs failing at least 3 jackknife analyses. Genes being driven by a single cohort in the meta-analysis (i.e. those failing exactly one jackknife test) represent potential false-positives. The addition of more cohorts to the meta-analysis should efficiently remove those that are false findings, as increasing the number of large *P*-values in the multiplication performed in Fisher’s method will increase the meta *P*-value. Moreover, adding more cohorts to the meta-analysis will increase the robustness of the results.

Some of the robust genes in this study have been previously identified as candidate genes for feed efficiency or as DEG associated with feed efficiency in livestock. For example, *CST6*, which passed all 5 jackknife tests for intake was also found to be differentially expressed in the muscle tissue of pigs with variation in RFI [31]. In addition, one of the genes that failed all 5 jackknife tests for intake was *LOC508916*, which is potentially a carboxylesterase 1-like gene. Two carboxylesterase genes (*CES1, CES3*) were identified as two of the most highly differentially expressed genes in the adipose tissue of pigs with low RFI [32].

One of the four genes identified in the interaction analysis was albumin (*ALB*). Plasma and serum levels of albumin have been associated previously with feed efficiency in steers and lambs. Paula et al. [33] demonstrated that the most efficient lambs had lower serum albumin concentrations. This phenomenon was also identified in the plasma of beef steers [34]. Another study by Connell et al. [35] showed a relationship between serum albumin levels and DMI in sheep. In this study, the transcript abundance of *ALB* is higher in animals with low gain and high intake. Our analysis included gain and intake phenotypes, suggesting that the level of albumin transcripts may either be influencing or responding to both phenotypes, rather than intake alone.

The gene *UQCRQ* was identified as being associated with gain. This gene had the same direction of expression in all five cohorts of animals (i.e., lower transcript abundance with higher gain). The *UQCRQ* gene is involved in mitochondrial energy production and has also been associated with feed efficiency in previous studies. Kong et al. [36] identified higher transcript abundance of *UQCRQ* in the rumen tissue of animals with low RFI. While it is difficult to cross compare these two phenotypes, it is of interest that this gene and the mitochondrial energy pathways were identified in both studies. One important detail to note about the *UQCRQ* gene is that it was one of the genes that failed only one of the jackknife tests, indicating potential cohort bias. As mentioned before, data from additional cohorts is needed to determine if this gene is indeed a robust biomarker of gain.

Recently, lower expression of *MYOZ2* was detected in the muscle of chickens with high feed efficiency [37], while another study found the expression of *MYOZ2* in a different population of chickens to be opposite in direction, with lower expression among birds with higher feed efficiency [38]. We found *MYOZ2* to be expressed in higher transcript abundance in four of the five cohorts of steers with higher gain. Similar to the two studies in which *MYOZ2* was associated with feed efficiency in chickens, this gene does not show 100% concordance in its direction of gene expression. The differences in expression studies in chickens were potentially attributed to differences in genetics or other factors [37]. In our study, there is some variation in breed representation among our phenotypic groups, but there is also variation in environment for each of the 5 cohorts, which may also be contributing to the variation in the direction of gene expression.

Most of the DEGs identified in our analysis exhibited variation in the direction of expression among cohorts. This supports the hypothesis that the expression of DEGs may be environmentally driven. It is also possible that other genes that were not significant in our differential expression analysis are involved in the regulation of these DEGs and pathways. Future work will focus on using gene expression profiles and clustering analysis to identify additional regulatory genes that may play a role in ADG and ADFI phenotypes.

## Conclusions

Data presented here demonstrate that finishing beef cattle with divergent ADFI and ADG phenotypes have gene expression differences that indicate that there are potentially differences in mitochondrial energy production and oxidative stress pathways, amino acid metabolism pathways, and cell signaling pathways. This work is a first step in integrating sequence data from multiple cohorts to identify potential biomarkers related to the gain and feed intake of beef cattle. Further study is needed to understand the role of natural variation in the skeletal muscle and its contribution to feed efficiency.

## Additional files

Additional file 1: (A) Cohort and age of animals selected for this study. (B) Breed percentages of phenotypic quadrants for each cohort (HH = high gain, high intake; LH = low gain, high intake; LL = low gain, low intake; HL = high gain, low intake). (C) Rationale for exclusion of animals within each cohort. (D) Sequencing statistics. (XLSX 27 kb)

Additional file 2: DEGs associated with the gain main effect in the individual cohort and meta analyses. Genes are ordered by adjusted meta-*P*-value. The individual cohort cells for DEGs identified in the meta-analysis are colored according to the sign of their log2 fold change, where green indicates up-regulation and red indicates down-regulation in high gain. Genes with all gray cell indicate those that were excluded because they were also significant for the gain by intake interaction term. (XLSX 1224 kb)

Additional file 3: DEGs associated with the intake main effect in the individual cohort and meta analyses. Genes are ordered by adjusted meta-*P*-value. The individual cohort cells for DEGs identified in the meta-analysis are colored according to the sign of their log2 fold change, where green indicates up-regulation and red indicates down-regulation in high intake. Genes with all gray cell indicate those that were excluded because they were also significant for the gain by intake interaction term. (XLSX 1241 kb)

Additional file 4: DEGs associated with the gain by intake interaction effect in the individual cohort and meta analyses. Genes are ordered by adjusted meta-*P*-value. The individual cohort cells for DEGs identified in the meta-analysis are colored according to the sign of their log2 fold change, where green indicates up-regulation and red indicates down-regulation in low gain, high intake. (XLSX 1218 kb)

Additional file 5: Jackknife sensitivity analysis results for the DEGs associated with the gain main effect. Genes with all gray cell indicate those that were excluded because they were also significant for the gain by intake interaction term. Jackknife 1 *P*-value gives the adjusted *P*-value for the meta-analysis with Cohort 1 removed, Jackknife 2 *P*-value gives the adjusted *P*-value for the meta-analysis with Cohort 2 removed, and so on. Yellow cells indicate jackknife analyses where the *P*-value was insignificant, i.e. the gene failed to pass the jackknife analysis. (XLSX 987 kb)

Additional file 6: Jackknife sensitivity analysis results for the DEGs associated with the intake main effect. Genes with all gray cell indicate those that were excluded because they were also significant for the gain by intake interaction term. Jackknife 1 *P*-value gives the adjusted *P*-value for the meta-analysis with Cohort 1 removed, Jackknife 2 P-value gives the adjusted P-value for the meta-analysis with Cohort 2 removed, and so on. Yellow cells indicate jackknife analyses where the *P*-value was insignificant, i.e. the gene failed to pass the jackknife analysis. (XLSX 1095 kb)

Additional file 7: Jackknife sensitivity analysis results for the DEGs associated with the gain by intake interaction. Jackknife 1 P-value gives the adjusted P-value for the meta-analysis with Cohort 1 removed, Jackknife 2 *P*-value gives the adjusted P-value for the meta-analysis with Cohort 2 removed, and so on. Yellow cells indicate jackknife analyses where the *P*-value was insignificant, i.e. the gene failed to pass the jackknife analysis. (XLSX 1030 kb)

## Abbreviations

ADFI: Average daily feed intake
ADG: Average daily gain
ANODEV: Analysis of deviance
DEG: Differentially expressed gene(s)
DMI: Dry matter intake
DNA: Deoxyribonucleic acid
FCR: Feed conversion ratio
GLM: Generalized linear model
GO: Gene ontology
IPA: Ingenuity pathway analysis
NCBI: National Center for Biotechnology Information
PANTHER: Protein analysis through evolutionary relationships
RFI: Residual feed intake
RNA: Ribonucleic acid
RNA-Seq: Ribonucleic acid sequencing
